# Dysregulated sexuality and childhood trauma in eating disorders: Psychopathological, biological, and behavioural correlates

**DOI:** 10.1192/j.eurpsy.2021.322

**Published:** 2021-08-13

**Authors:** G. D’Anna, G. Castellini, E. Rossi, E. Cassioli, C. Appignanesi, A.M. Monteleone, A.H. Rellini, V. Ricca

**Affiliations:** 1 Department Of Health Sciences, University of Florence, Florence, Italy; 2 Department Of Psychiatry, University of Campania “Luigi Vanvitelli”, Naples, Italy; 3 Department Of Psychological Science, University of Vermont, Burlington, United States of America

**Keywords:** hypersexuality, childhood trauma, eating disorders, emotion dysregulation

## Abstract

**Introduction:**

Sexual dysfunction is common in eating disorders (EDs), but its relevance is often overlooked.

**Objectives:**

To describe different ED clinical subgroups in terms of psychopathology, putative biological correlates, and consequences of dysregulated sexuality, focusing on the role of childhood trauma.

**Methods:**

Healthy controls (n=60), binge-purging (n=38), and restricting patients (n=24) were compared (age- and BMI-adjusted ANOVA; Bonferroni post-hoc tests), using total scores of Eating Disorder Examination Questionnaire (EDE-Q), Emotional Eating Scale (EES), SCL-90-R Global Severity Index (GSI), Barratt Impulsiveness Scale (BIS-11), Difficulties in Emotion Regulation Scale (DERS), Childhood Trauma Questionnaire (CTQ), Female Sexual Functioning Index (FSFI), Hypersexual Behaviour Inventory (HBI), and patients’ hormonal profiles (gonadal and pituitary hormones, ghrelin). Self-reported voluntary termination of pregnancy (VTP) and promiscuous sexual activity were recorded. For ED patients (N=62), regression analyses between significant variables and HBI were carried, applying moderation models for different CTQ scores.

**Results:**

Table 1 outlines significant between-group comparisons (°: different from controls; *: different from restricting patients; p<0.05). Binge-purging patients had higher FSH, LH, and ghrelin levels, more VTPs and promiscuity. HBI showed significant correlations with EES, SCL-90-R-GSI, DERS, CTQ, and ghrelin levels. CTQ moderated interactions for DERS and EES (Figure 1).

**Table 1:** 

	Binge-purging	Restricting	Controls	F
EDE-Q	3.86±1.20°	3.41±1.64°	0.85±0.83	67.32
EES	40.85±22.74°*	16.01±15.88	19.87±15.21	7.01
SCL-90-R GSI	1.73±0.65°	1.27±0.69°	0.68±0.44	20.32
BIS-11	62.47±9.91°	60.81±8.56	57.04±10.04	4.99
DERS	106.97±29.15°*	83.97±33.12	78.14±14.12	10.21
CTQ	55.32±21.06°	49.31±10.81°	38.02±8.32	15.24
FSFI	17.32±11.89°*	11.70±10.98°	29.32±7.45	24.02
HBI	28.75±13.89*	20.56±3.12	26.11±4.90	4.92

**Figure:**
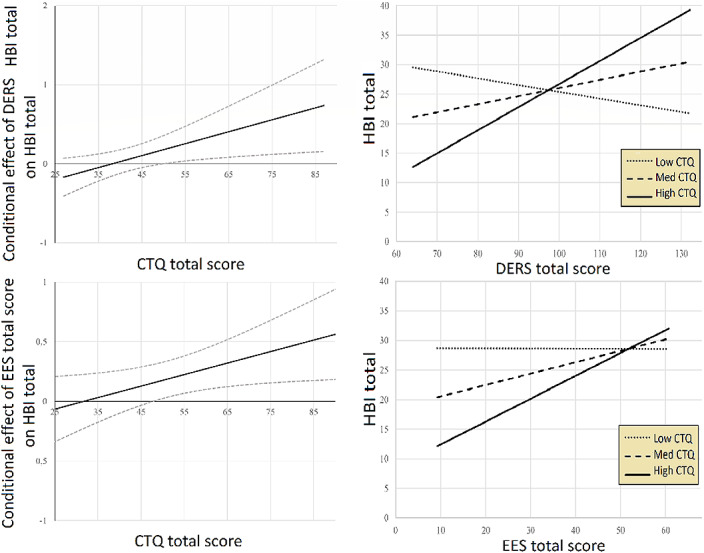

**Conclusions:**

Dysregulated sexuality is linked to emotion dysregulation and childhood trauma. Binge-purging patients experience adverse behavioural consequences.

**Disclosure:**

No significant relationships.

